# Efficacy of typhoid conjugate vaccine in Nepal: final results of a phase 3, randomised, controlled trial

**DOI:** 10.1016/S2214-109X(21)00346-6

**Published:** 2021-10-19

**Authors:** Mila Shakya, Merryn Voysey, Katherine Theiss-Nyland, Rachel Colin-Jones, Dikshya Pant, Anup Adhikari, Susan Tonks, Yama F Mujadidi, Peter O’Reilly, Olga Mazur, Sarah Kelly, Xinxue Liu, Archana Maharjan, Ashata Dahal, Naheeda Haque, Anisha Pradhan, Suchita Shrestha, Manij Joshi, Nicola Smith, Jennifer Hill, Jenny Clarke, Lisa Stockdale, Elizabeth Jones, Timothy Lubinda, Binod Bajracharya, Sabina Dongol, Abhilasha Karkey, Stephen Baker, Gordan Dougan, Virginia E Pitzer, Kathleen M Neuzil, Shrijana Shrestha, Buddha Basnyat, Andrew J Pollard

**Affiliations:** a Oxford University Clinical Research Unit, Patan Academy of Health Sciences, Lalitpur, Nepal; b Oxford Vaccine Group, Department of Paediatrics, University of Oxford, Oxford, UK; c NIHR Oxford Biomedical Research Centre, Oxford, UK; d Patan Academy of Health Sciences, Patan Hospital, Lalitpur, Nepal; e Nepal Family Development Foundation, Lalitpur, Nepal; f Wasa Pasa Polyclinics Private, Lalitpur, Nepal; g Centre for Tropical Medicine and Global Health, University of Oxford, Oxford, UK; h Cambridge Institute of Therapeutic Immunology & Infectious Disease, University of Cambridge, Cambridge, UK; i Department of Epidemiology of Microbial Diseases, Yale School of Public Health, Yale University, New Haven, CT, USA; j School of Medicine, University of Maryland, Baltimore, MD, USA

## Abstract

**Background:**

Typhoid fever is a major public health problem in low-resource settings. Vaccination can help curb the disease and might reduce transmission. We have previously reported an interim analysis of the efficacy of typhoid conjugate vaccine (TCV) in Nepali children. Here we report the final results after 2 years of follow-up.

**Methods:**

We did a participant-masked and observer-masked individually randomised trial in Lalitpur, Nepal, in which 20 019 children aged 9 months to younger than 16 years were randomly assigned in a 1:1 ratio to receive a single dose of TCV (Typbar TCV, Bharat Biotech International, India) or capsular group A meningococcal conjugate vaccine (MenA). Participants were followed up until April 9, 2020. The primary outcome was blood culture-confirmed typhoid fever. Cases were captured via passive surveillance and active telephone surveillance followed by medical record review. The trial is registered at ISRCTN registry, ISRCTN43385161 and is ongoing.

**Findings:**

From Nov 20, 2017, to April 9, 2018, of 20 119 children screened, 20 019 participants were randomly assigned to receive TCV or MenA vaccine. There were 75 cases of blood culture-confirmed typhoid fever included in the analysis (13 in the TCV group and 62 in the MenA group) over the 2-year period. The protective efficacy of TCV against blood culture-confirmed typhoid fever at 2 years was 79·0% (95% CI 61·9–88·5; p<0·0001). The incidence of typhoid fever was 72 (95% CI 38–123) cases per 100 000 person-years in the TCV group and 342 (95% CI 262–438) cases per 100 000 person-years in the MenA group. Adverse events occurring within the first 7 days post-vaccination were reported previously.

**Interpretation:**

The final results of this randomised, controlled trial are in keeping with the results of our published interim analysis. There is no evidence of waning protection over a 2-year period. These findings add further support for the WHO recommendations on control of enteric fever.

**Funding:**

Bill & Melinda Gates Foundation.

## Introduction

Typhoid fever, once endemic in high-income countries, remains a major public health concern in low-resource settings where there is inadequate sanitation and poor hygiene conditions, including Nepal.^1^, ^2^, ^3^ More than 9 million cases of febrile illnesses and 110 000 deaths are attributed to typhoid fever each year.^4^ South Asia accounts for over 70% of the global burden.^1^ Growing antimicrobial resistance in the region, especially outbreaks of cephalosporin-resistant typhoid in Pakistan, poses a challenge in the treatment of typhoid fever.^5^, ^6^

Access to clean drinking water, adequate sanitation and good hygiene are essential for control of typhoid fever, but the substantial costs associated with the large infrastructure changes and difficulties in making and maintaining behavioural changes in low-resource settings has been challenging. Vaccines against typhoid fever can be a cost-effective interim solution to curb the disease and antimicrobial resistance associated with it.^7^, ^8^ Although licensed vaccines are available, their use has been limited to travellers. The parenteral Vi polysaccharide vaccines are poorly immunogenic in young children. The oral Ty21a vaccine is not licensed for children younger than 6 years and is difficult for most children to swallow. Vi-rEPA, a typhoid conjugate vaccine (TCV), showed high efficacy in young children; however, never advanced to market approval.^9^ More recently, a new TCV containing a Vi polysaccharide conjugated to tetanus-toxoid protein carrier (Typbar-TCV) has shown high immunogenicity and the ability to elicit immunological responses in young children.^10^ In principle, TCVs can elicit a longer duration of protection in comparison with polysaccharide vaccines.^9^ The WHO Strategic Advisory Group of Experts, in October, 2017, recommended the use of TCV for the control of endemic and epidemic typhoid fever and in December, 2017, Typbar-TCV was WHO-prequalified.^11^

**Research in context**

**Evidence before this study**

We searched PubMed for research articles on typhoid conjugate vaccine efficacy in children published any time before April 2, 2020, with no language restriction. We used the search terms “typhoid fever”, “conjugate vaccine”, “high burden”, and “children”. A randomised, controlled trial of Vi-rEPA, a conjugate vaccine containing Vi bound to non-toxic recombinant *Pseudomonas aeruginosa* exotoxin A, done in Vietnam in children aged 2–5 years showed a vaccine efficacy of over 90% over 27 months of follow-up. The vaccine, however, is unavailable in the market.The interim analysis of this study has been published previously in which we reported that a single dose of typhoid conjugate vaccine (TCV) given to children aged 9 months to younger than 16 years conferred over 80% protection in the first 12 months after vaccination. There are no other published studies showing the efficacy of this typhoid conjugate vaccine.

**Added value of this study**

This study shows the protective efficacy of the TCV in children over 2 years and supplements the previously reported one-year interim results of this study. Additionally, we report the immunogenic response to the vaccine up to 18 months post-vaccination. The results suggest that a 4-fold increase in antibody titre is maintained in a large proportion of the children at 18 months.

**Implications of all the available evidence**

The results of the study show that protection is maintained with a single dose of typhoid conjugate vaccine protection up to 2 years after vaccination. The results support the WHO recommendations to vaccinate children in endemic typhoid settings. Further data on medium-term to long-term efficacy of the vaccine is needed to establish the need for booster doses.

We did a phase 3 randomised, controlled trial of a single dose of TCV in Lalitpur, Nepal, where typhoid is endemic, as a part of the Typhoid Vaccine Acceleration Consortium (TyVAC),^12^ with the aim of establishing the field efficacy of TCV. TyVAC is a partnership between the Center for Vaccine Development and Global Health at the University of Maryland School of Medicine, the Oxford Vaccine Group at the University of Oxford, and PATH, an international non-profit organisation. In 2019, we reported the interim results after 1 year of follow-up from the ongoing trial.^13^ A single dose of TCV had an efficacy of 81·6% (95% CI 58·8–91·8; p<0·001) in the first year after vaccination. Seroconversion defined as a 4-fold increase in anti-Vi IgG titres 28 days post-vaccination was 99% in the TCV group. We now report the final efficacy results after 2 years of follow-up.

## Methods

### Study design and participants

In this phase 3, double-blind, individually randomised, controlled trial in Lalitpur Metropolitan City, an urban area within Kathmandu Valley, Nepal, we enrolled children aged 9 months to younger than 16 years old. Parents or legal guardians provided written informed consent before enrolment, and assent was sought from children aged 7 years and older. Follow-up of the trial participants was planned for a 2-year period until the last participant was unmasked. The unmasking process started from Jan 20, 2020. Each participant was offered the alternative vaccine to the one they previously received, and a blood sample was taken in a subset of participants. On March 22, 2020, after the government of Nepal's announcement to restrict all unnecessary movement of people owing to the COVID-19 pandemic, the field activities were stopped and unmasking and revaccination could not be completed. The study participants were informed about the suspension of the vaccination and will be unmasked and offered vaccination after the situation improves and in accordance with government directives. Unmasking and vaccination was resumed from Jan 25, 2021. After being temporarily suspended again on April 28, 2021, owing to a second wave of COVID-19, the study has again resumed from May 27, 2021. Passive surveillance clinics remained open for the 2-year period until April 9, 2020, providing a full 2 years of follow-up for the primary efficacy endpoint.

The trial was reviewed and approved by the Oxford Tropical Research Ethics Committee and the Nepal Health Research Council and was done in accordance with the principles of the Declaration of Helsinki. The protocol has previously been published and the details of the inclusion and exclusion criteria are included in the appendix (p 3).^13^, ^14^

### Randomisation and masking

Participants were enrolled and randomly assigned during the vaccination visit by means of 1:1 stratified block randomisation, with random block sizes of 6–12 by means of an offline mobile application built for the purpose. The participants received a single 25 μg/0·5 mL dose of tetanus-toxoid conjugated Vi polysaccharide typhoid vaccine (Typbar TCV; Bharat Biotech International, India) or meningococcal capsular group A conjugate vaccine (MenA; MenAfriVac, Serum Institute of India, Pune, India). In addition, from a subgroup of consenting participants, 1500 were randomly selected (1000 in the TCV group and 500 in the MenA group) for the immunogenicity component of the study. Only staff administering the vaccine were aware of which vaccine was administered and these staff were not involved in the follow-up of participants.

### Procedures

Blood cultures were obtained from participants presenting to the trial clinics (Patan Hospital, Lalitpur or 18 community-based clinics) with reported fever for at least 2 full days or a current temperature of at least 38°C. Blood cultures were done at Patan Hospital following the standard operating procedures. Additional participants who attended health facilities other than the trial clinics were identified from routine tri-monthly phone calls to participants. Medical records of these participants were reviewed for blood culture-confirmed diagnosis. Only participants with confirmed blood culture-positive typhoid fever are included in the primary efficacy analysis.

Following vaccination, parents and guardians were asked to inform the study doctors about any adverse events. All adverse events, occurring within the first 7 days post-vaccination, were recorded via phone call and have been described and reported previously. Serious adverse events in the 2-year study period were identified from participants visiting the study clinics, surveillance at Patan Hospital, and via tri-monthly phone calls. Serious adverse events were followed up and recorded by study doctors. Any serious adverse events were recorded on a tri-monthly basis.

Blood sample collection was planned on day 0, day 28, 18 months, and 24 months post-vaccination. Blood samples were temporarily stored at 2–8°C, processed within 6 h of blood draw and stored at −20°C. We were able to complete 18 months sample collection, with 381 participants bled at 24 months at the unmasking visit. Further sample collection at 24 months is pending as the study activities were halted owing to the COVID-19 pandemic.

Anti-Vi IgG and IgA titres at day 0, day 28, and 18 months were measured in plasma samples at the Oxford Vaccine Group Laboratory, University of Oxford. The overall day 0 and day 28 anti-Vi IgG results have been previously reported.^13^ Anti-Vi IgG titres were measured by means of a commercial ELISA kit (VaccZyme, The Binding Site, Birmingham, UK) according to the manufacturer guidelines. Anti-Vi IgA titres were assessed with Vi-coated plates and reagents supplied by The Binding Site by means of a protocol adapted from the commercial VaccZyme assay.

### Outcomes

The primary outcome was incidence of blood culture-confirmed typhoid fever. The secondary outcomes were incidence of blood culture-confirmed typhoid fever in participants with at least 3 full days of fever; incidence of blood culture-confirmed paratyphoid fever; numbers of clinically suspected typhoid fever; numbers and duration of inpatient or outpatient fever; and length of hospital admission. The exploratory outcomes were absenteeism from school or work as the result of illnesses; and all-cause mortality. All exploratory outcomes were prespecified. Immunogenicity of TCV at 1 month post-vaccination and persistence of antibodies induced by TCV at 18 and 24 months were explored.

### Statistical analysis

A target sample size of 20 000 children was required in the trial, and under the assumptions of the power calculation approximately 36 cases of typhoid fever in the control group and nine cases in the TCV group were expected to be observed (see appendix p 2). The primary outcome, the incidence of blood culture-confirmed typhoid fever, was calculated as the number of cases divided by the total number of person-years of follow-up. Only cases occurring more than 14 days after vaccination were included in the analyses. The vaccine efficacy was calculated as (1 − IRR) × 100%, where IRR is the incidence rate ratio (TCV:MenA), estimated by means of a Poisson regression model. Prespecified subgroup analyses of the primary outcome were done by means of Poisson models with interaction effects.

The cumulative incidence of typhoid was summarised by means of the Kaplan-Meier method. For any participant with more than one blood culture-confirmed typhoid fever, only the first event was used in the Kaplan-Meier analysis, but all events were included in incidence estimates.

Numbers and proportions of the secondary and exploratory outcomes were summarised. p values less than 0·05 were considered significant. Seroconversion, defined as a 4-fold increase in antibody titre from baseline, was assessed at day 28 and 18 months post-vaccination

This trial is registered with the ISRCTN registry, ISRCTN43385161.

### Role of the funding source

The funder had no role in the study design, data collection, data analysis, data interpretation, or writing of the report.

## Results

From Nov 20, 2017, to April 9, 2018, a total of 20 119 children underwent screening. Of 20 019 children enrolled in the trial, 10 005 were randomly assigned to the TCV group and 10 014 to the MenA group (appendix p 4). Table 1 shows the characteristics of the participants, which were similar in the two groups of the trial.

**Table 1:** Baseline characteristics

	**Typhoid conjugate vaccine group (n=10 005)**	**Group A meningococcal vaccine group (n=10 014)**
**Sex**		
Female	4899 (48·97%)	4856 (48·49%)
Male	5106 (51·03%)	5158 (51·51%)
**Age, years**		
<2	671 (6·71%)	719 (7·18%)
2 to <5	2990 (29·89%)	2964 (29·60%)
≥5 years	6344 (63·41%)	6331 (63·22%)

Data are n (%).

During the follow-up period, between Dec 6, 2017, and April 9, 2021, 3433 blood cultures were done. There were 76 cases of blood culture-confirmed typhoid fever, of which 58 were identified through passive surveillance and 18 through medical records review. One case occurred within 14 days of vaccination and was excluded from the analyses. One participant in the MenA group had two episodes of culture-positive typhoid fever. In the first episode, the 8-year-old male presented with upper respiratory tract symptoms and was treated for the same. The blood culture was positive for typhoid fever. 3 months after the first presentation, the participant presented with similar symptoms and was diagnosed with and had culture-positive typhoid fever.

There were 13 cases of culture-positive typhoid fever in the TCV group and 62 cases in the MenA group, giving a protective efficacy of 79·0% (95% CI 61·9–88·5%; p<0·0001; figure and table 2). There were 64 isolates available for antimicrobial susceptibility testing (appendix p 19) of which 43 (81%) of 53 isolates in the MenA group and ten (91%) of 11 in the TCV group were non-susceptible to ciprofloxacin. Two isolates, one in each group, had reduced susceptibility to azithromycin. There were no multidrug resistant strains and no strains resistant to ceftriaxone.

**Figure:**
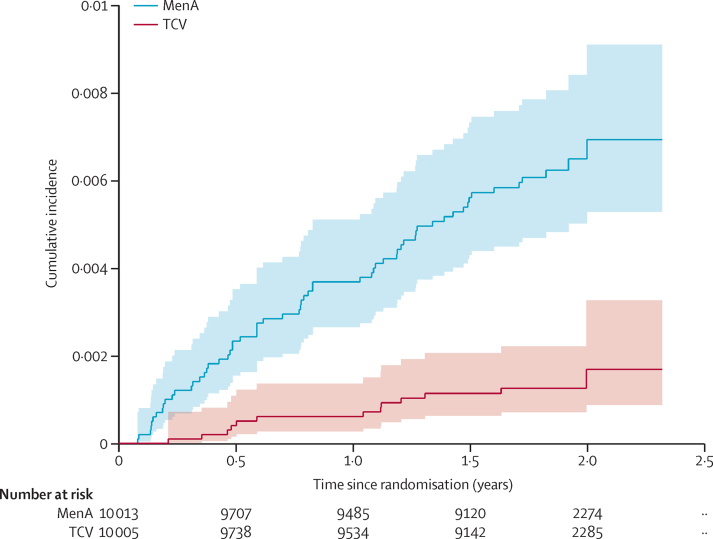
Kaplan-Meier estimates of the cumulative incidence of blood culture confirmed typhoid fever, according to trial group Blood culture-positive typhoid fever was the primary outcome. TCV=Typhoid conjugate vaccine. MenA=group A meningococcal vaccine (control).

**Table 2:** Occurrence of blood culture-confirmed typhoid and paratyphoid fever and protective efficacy of typhoid conjugate vaccine

	**Typhoid conjugate vaccine group (n=10 005)**	**Incidence per 100 000 person-years (95% CI)**	**Group A meningococcal vaccine group (n=10 014)**	**Incidence per 100 000 person-years (95% CI)**	**Vaccine efficacy (95% CI)**	**p value**
Person-years of follow-up*	18 145	..	18 154	..	..	..
Blood culture-confirmed typhoid fever in first 14 days after vaccination	..	..	1	..	..	..
Blood culture-confirmed typhoid fever after 14 days†	13	72 (38 to 123)	62	342 (262 to 438)	79·0% (61·9 to 88·5)	<0·0001
Detected through fever clinics	11 (84·6%)	..	47 (75·8%)	..	..	..
Detected through active follow-up and medical record review	2 (15·4%)	..	15 (24·2%)	..	..	
Blood culture-confirmed typhoid fever after 14 days (one case per person)	13	72 (38 to 123)	61	336 (258 to 432)	78·7% (61·2 to 88·3)	<0·0001
Blood culture-confirmed typhoid fever in those with ≥3 days of fever before blood culture (fever clinics)‡	4	22 (6 to 57)	30	166 (112 to 237)	86·7% (62·2 to 95·3)	<0·0001
Blood culture-confirmed paratyphoid fever after 14 days§	7	39 (15 to 79)	6	33 (12 to 72)	−16·7% (−247·4 to 60·8)	0·78

Data are n (%) unless stated otherwise.

* Participants with no follow-up contact contribute half a day follow-up in calculations.

† For all reported culture-positive cases reported from medical records review, isolates were checked, when available, to reconfirm diagnostic results.

‡ From fever clinic cases only; 17 cases detected through medical records review were excluded as data on number of days of fever was not available.

§ One case on day 3 post-vaccination excluded from TCV group.

A total of 34 participants with blood culture-confirmed typhoid were febrile for at least 3 completed days before the blood culture, and the vaccine efficacy in this subgroup was 86·7% (95% CI 62·2–95·3; p<0·0001). There were two participants with blood culture-confirmed typhoid in the under-2-year age group, both in the TCV group (vaccine efficacy not computed), 19 in the 2 to younger than 5-year age group (vaccine efficacy 64·9%, 95% CI 2·7–87·4) and 53 in those aged 5 years and older (vaccine efficacy 87·5%, 95% CI 71·0–94·7; table 3). There was no significant difference in vaccine efficacy between age groups (2 to <5 years *vs* <5 years; p=0·98). The protective efficacy of TCV was 83·4% (95% CI 60·5–93·0) in the first 12 months of follow-up compared with 73·0% (95% CI 37·9–88·3) after the first year (p=0·43 for the difference in vaccine efficacy between year 1 and year 2; table 3).

**Table 3:** Subgroup comparison of protective efficacy of typhoid conjugate vaccine

		**Typhoid conjugate vaccine group (n=10 005)**	Incidence per 100 000 person-years*(95% CI)	**Group A meningococcal vaccine group (n=10 014)**	**Incidence per 100 000 person-years (95% CI)**	**Vaccine efficacy (95% CI)**	**p value (interaction)**
Age, years		..	..	..	..	..	0·13†
	<2	2	167 (20– 604)	0	..	..	..
	2 to <5	5	127 (41–296)	14	362 (198–607)	64·9% (2·5–87·3)	..
	≥5	6	46 (17–100)	47	369 (272–489)	87·5% (70·8–94·5%)	..
Sex		..	..	..	..	..	0·12
	Male	5	54 (18–126)	39	417 (297–570)	87·0% (67·1–94·9)	..
	Female	8	90 (39–180)	23	262 (166–392)	65·6% (23·1–84·6)	..
Time since vaccination, months		..	..	..	..	..	0·43
	≤12	6	61 (23–134)	36	370 (259–512)	83·4% (60·5–93·0)	..
	>12	7	83 (34–172)	26	310 (202–454)	73·0% (37·9– 88·3%)	..

Data are n (%) unless stated otherwise.

* Blood culture confirmed typhoid fever after 14 days.

† p value for interaction inclusive of 2 to <5 years and 5 years and older.

There were 13 cases of blood culture-confirmed paratyphoid fever, seven in the TCV group and six in the MenA group (vaccine efficacy −16·7%, 95% CI −247·4 to 60·8%; p=0·78).

Table 4 shows the anti-Vi IgG and IgA response in the TCV group and MenA group. At 28 days post-vaccination, 664 (97%) of 683 in the TCV group and six (2%) of 380 in the MenA group had Vi-IgA seroconversion, whereas 677 (99%) of 683 in the TCV group and eight (2%) of 380 in the MenA group had anti-Vi IgG seroconversion (as previously reported). At 18 months post-vaccination, 479 (89%) of 539 in the TCV group and six (2%) of 299 in the MenA group had Vi-IgA seroconversion, whereas 573 (95%) of 601) in the TCV group and ten (3%) of 331 in the MenA group had Vi-IgG seroconversion.

**Table 4:** Anti-Vi IgA and IgG titres at baseline, 28 days, and 18 months after randomisation in the immunogenicity cohort

		**Day 0**	**Day 28**	**Month 18**	**Day 0–day 28**	**Day 0–month 18**
**Anti-Vi IgA titre**						
Typhoid conjugate vaccine group						
	Level above lower limit of quantification of the assay*	56/683 (8%)	670/683 (98%)	601/639 (94%)	664/683 (97%; 4-fold increase)	479/539 (89%; 4-fold increase)
	Geometric mean concentration (95% CI), EU per mL	1·79 (1·72 to 1·87)	122·04 (111·83 to 133·17)	22·86 (20·81 to 25·12)	120·25 (110·59 to 129·90)-fold increase	27·50 (24·54 to 30·46)-fold increase
	Median (IQR)	1·56 (1·56 to 1·56)	136·54 (66·74 to 273·81)	22·60 (11·30 to 50·65)	..	..
Group A meningococcal vaccine group						
	Level above lower limit of quantification of the assay*	25/380 (7%)	38/380 (10%)	41/358 (11%)	6/380 (2%; 4-fold increase)	6/299 (2%; 4-fold increase)
	Geometric mean concentration (95% CI), EU per mL	1·74 (1·67 to 1·83)	1·87 (1·75 to 1·99)	1·85 (1·75 to 1·96)	1·79 (1·09 to 2·49)-foldincrease	1·27 (1·10 to 1·43)-foldincrease
	Median (IQR)	1·56 (1·56 to 1·56)	1·56 (1·56 to 1·56)	1·56 (1·56 to 1·56)	..	..
**Anti-Vi IgG titre**						
Typhoid conjugate vaccine group						
	Level above lower limit of quantification of the assay†	268/849 (32%)	708/709 (100%)	633/639 (99%)	677/683 (99%; 4-fold increase)	573/601 (95%; 4-fold increase)
	Geometric mean concentration (95% CI), EU per mL	7·21 (6·69 to 7·11)	2037·90 (1904·64 to 2180·48)	241·29 (220·23 to 264·36)	501·34 (463·67 to 539·01)-fold increase	67·39 (60·39 to 74·39)-fold increase
	Median (IQR)	3·7 (3·7 to 13·4)	2220·7 (1298·6 to 3725·7)	241·29 (220·24 to 264·36)	..	..
Group A meningococcal vaccine group						
	Level above lower limit of quantification of the assay†	112/460 (24%)	112/388 (29%)	96/358 (27%)	8/380 (2%; 4-fold increase)	10/331 (3%; 4-fold increase)
	Geometric mean concentration (95% CI), EU per mL	6·48 (5·89 to 7·13)	6·98 (6·20 to 7·85)	6·57 (5·87 to 7·35)	5·59 (−0·95 to 12·14)-foldincrease	1·52 (1·00 to 2·05)-foldincrease
	Median (IQR)	3·7 (3·7 to 8·9)	3·7 (3·7 to 10·5)	3·7 (3·7 to 9·40)	..	..

Data are n/N (%) unless stated otherwise.

* The lower limit of quantification was 3·125 EU per mL. Values below this limit were substituted with 1·56 EU per mL for the analysis.

† The lower limit of quantification was 7·4 EU per mL. Values below this limit were substituted with 3·7 EU per mL for the analysis. EU=ELISA units.

The anti-Vi IgA and IgG responses according to age group are shown in the appendix (pp 7–9). By day 28, in the TCV group, 52 (95%) of 55 participants younger than 5 years had seroconverted to Vi IgA, 221 (98%) of 225 participants aged from 5 to younger than 10 years had seroconverted to Vi IgA, and 391 (97%) of 403 participants aged 10 years and older had seroconverted to Vi IgA , whereas by 18 months these percentages had dropped to 25 (60%) of 42, 160 (88%) of 182, and 294 (93%) of 315, respectively. Similarly, the proportion with Vi-IgG seroconversion at day 28 was 54 (98%) of 55 for participants aged less than 5 years, 225 (100%) of 225 for those aged from 5 to younger than 10 years, and 342 (100%) of 343 for those aged 10 years and older in the TCV group, and remained high at 55 (95%) of 58, 202 (97%) of 209, and 316 (95%) of 334 by 18 months, respectively.

The anti-Vi IgG and anti-Vi IgA response according to sex are presented in the appendix (pp 10–13). The geometric mean concentration of anti-Vi IgA at 18 months was 19·70 (95% CI 17·30–22·43) ELISA units (EU) per mL in males and 26·96 (95% CI 23·56–30·86) in females. Males had a 25·20-fold (95% CI 21·28–29·12) increase in anti-Vi IgA titre and a 60·80-fold (95% CI 52·26−69·35) increase in anti-Vi IgG titre whereas females had a 29·93-fold (95% CI 25·46–34·40) increase in anti-Vi IgA titre and 74·60-fold (95% CI 63·31–85·89) increase in anti-Vi IgG titre. Females had significantly higher titre at 18 months (p=0·0016 for anti-Vi IgA and p=0·0080 for anti-Vi IgG) and significantly higher fold increase in anti-Vi IgA (p=0·033) and the anti-Vi IgG titres (p=0·031) from day 0 to 18 months post-vaccination in the TCV group.

The number of fever presentations at the study clinics and the self-reported fevers from follow-up phone calls, and the corresponding vaccine efficacies is shown in the appendix (p 14). There were 2352 fever cases in the TCV group and 2366 fever cases in the MenA group, which met the study criteria for blood culture and presented to the study clinics (vaccine efficacy 0·5%, 95% CI −5·3 to 6·1; p=0·85), of which 417 in the TCV group compared with 415 in the MenA group were clinically suspected to be typhoid fever (vaccine efficacy −0·5%, 95% CI −15·4 to 12·4; p=0·94). There were 4816 self-reported febrile episodes in the TCV group and 4766 in the MenA group (vaccine efficacy −1·1%, 95% CI −5·3 to 2·9%; p=0·59). The median duration of fever per episode was 3·0 days (IQR 2–4) in both groups. A total of 31 fever episodes were self-reported as clinically diagnosed typhoid in the TCV group, whereas 71 were reported in the MenA group (vaccine efficacy 56·3%, 95% CI 32·5–72·3; p<0·0001).

A total of 149 children self-reported to be admitted to hospital in the TCV group compared with 141 in the MenA group (vaccine efficacy −5·7%, 95% CI −34·1 to 16·6; p=0·64; appendix p 16). Both groups self-reported 5 days (IQR 3–7) of hospital stay. There were eight deaths, three in the TCV group and five in the MenA group over the study period. None of the deaths were vaccine-related (appendix p 18).

The self-reported days missed from school and work are shown in the appendix (pp 16–17). There were 1928 children who missed school in the TCV group compared with 1859 children in the MenA group, both groups with a median 4 days (IQR 2–7) of missed school. Among those self-reporting typhoid fever, there were 28 episodes in the TCV group and 61 episodes in the MenA group with median 10 days (IQR 5–14·5) and 10 days (IQR 6–15) of school missed, respectively.

## Discussion

Among a cohort of Nepali children, the protective efficacy of single dose TCV against blood culture-positive typhoid fever in 2 years post-vaccination is similar to that reported in the interim efficacy analysis (vaccine efficacy 81·6%, 95% CI 58·8–91·8; p<0·001).^13^ There was also no significant difference in the vaccine efficacy during the first 12 months of follow-up versus thereafter. The vaccine efficacy is also similar to seroefficacy estimates (vaccine efficacy 85%, 95% CI 80–88) from a phase 3 immunogenicity trial in India.^15^

Immunogenicity of TCV was robust in this population. Our results showed a 99% seroconversion of anti-Vi IgG antibodies and a 97% seroconversion of anti-Vi IgA antibodies at day 28. Similar anti-Vi IgG immune responses to TCV have previously been reported in studies from Vietnam and India.^9^, ^10^ In a study evaluating the antibody response to Vi-polysaccharide and Vi-conjugate vaccines in a typhoid fever controlled human infection model, there were high anti-Vi IgG and IgA responses to TCV, and anti-Vi IgA antibodies were associated with protection in individuals who received TCV.^16^ Further, sustained 95% and 89% seroconversion at 18 months shows persistence of immunological responses.

There were significant differences in the increase in anti-Vi IgG and IgA titres from baseline to day 28 as well as from baseline to 18 months post-vaccination across age groups, with the increse being lowest in the youngest age group. There was no significant difference in efficacy between the 2 to younger than 5 year and 5 year and older age groups or by time since vaccination. The presence of comparatively high titre of antibodies at baseline in the older age groups is suggestive of environmental exposure to typhoid and, as a result, some degree of pre-existing protective immunity against the disease. This might imply that some of the differences in antibody titres by age result from boosting of pre-existing immunity rather than immaturity of the immune response in younger age groups. It has been suggested that infants produce lower and less persistent antibodies in response to T-cell dependent protein or conjugate vaccines, and maternal antibodies might suppress the response to vaccination, which could explain the results.^17^ However, a cluster randomised trial done in Dhaka, Bangladesh has shown high immunogenic response in children younger than 2 years,^18^ This result suggests that the small number of participants in the youngest age group of the immunogenicity component of our study limits interpretation of this finding. Similarly, there were only two cases of culture-positive typhoid fever in the younger than 2-years age group leading to a lack of power to establish vaccine efficacy in the youngest children in our study. However, other studies report robust efficacy in this age group, including a cluster randomised trial in Bangladesh showed consistent direct protection across all age groups including in those younger than 2 years of age.^18^

TCV did not reduce all-cause fever, hospital admissions, or absenteeism. TCV did significantly reduce clinically diagnosed typhoid cases, however the vaccine efficacy was lower than in culture-confirmed cases. In addition, TCV did not protect against paratyphoid fever, as expected; a separate vaccine against paratyphoid might be required.^19^ Given the non-specific symptoms associated with enteric fever, it is not surprising that not all clinically suspected cases would be true typhoid fever. It is difficult to distinguish typhoid from other diseases, including upper respiratory tract infections and typhus fevers in our setting.^20^, ^21^ The duration of fever in the TCV group, which was shorter than in the MenA group (6 days *vs* 7·5 days; p=0·0084), suggests that even in those with the disease, severity might be less after TCV administration.

A large proportion of the isolates were non-susceptible to ciprofloxacin. Azithromycin was used as a standard for treatment of suspected and diagnosed typhoid fever in the study. However, fluoroquinolones, including ciprofloxacin, are unfortunately still incorrectly being used in south Asia. No multidrug resistant strains or extensively drug-resistant strains were identified in the study, although extensively drug-resistant strains have been reported to cause epidemic in Pakistan. TCV will not only prevent typhoid fever, as suggested by our study, but might help in the control of antimicrobial resistance.^22^

Our data from 2 years of follow-up show that there is a high incidence of typhoid fever in the population (342 cases per 100 000 person-years in the MenA group). Safety of this vaccine has been previously shown.^13^ Model-based studies have shown typhoid burden in Nepal ranging from 297 (95% CI 128–472)^1^ to 449 (95% CI 383–521) per 100 000 person-years.^7^ Collectively, with a typhoid fever incidence of greater than 500 per 100 000 person-years,^1^ south Asia remains the hub for typhoid fever. Introduction of TCV in children in high-burden settings such as south Asia, as recommended by the WHO,^11^ might considerably reduce the burden of the disease as shown by this study.

Our data support the introduction of TCV in typhoid endemic settings. In south Asia, Pakistan is the only country administering TCV to its population.^23^ Other countries in south Asia, including Nepal (and other high-burden settings), should follow suit.

## Data sharing

De-identified individual participant data from this study will be made available to researchers without restriction when the study is complete. Data can be obtained by contacting merryn.voysey@paediatrics.ox.ac.uk. The statistical analysis plan and protocol are published.^13^, ^14^

## Declaration of interests

AJP is Chair of the UK Department of Health and Social Care's (DHSC) Joint Committee on Vaccination & Immunisation (JCVI) and is a member of WHO's Strategic Advisory Group of Experts on Immunization. AJP is a National Institute for Health Research (NIHR) Senior Investigator. The views expressed in this article do not necessarily represent the views of DHSC, JCVI, NIHR, or WHO. AJP is chief investigator on clinical trials of Oxford University's COVID-19 vaccine funded by NIHR. Oxford University is in a joint COVID-19 vaccine development partnership with AstraZeneca. VEP is a member of the WHO Immunization and Vaccine-related Implementation Research Advisory Committee and has received reimbursement from Merck and Pfizer for travel expenses to attend scientific input engagements unrelated to typhoid vaccines. All other authors declare that they have no conflict of interest.
