# Supplementary material for: Efficacy of typhoid conjugate vaccine in Nepal: final results of a phase 3, randomised, controlled trial
Source: Lancet Glob Health. 2021 Oct 19;9(11):e1561–8. doi: 10.1016/S2214-109X(21)00346-6 (PMC8551681; doi:10.1016/S2214-109X(21)00346-6)
Supplement: Supplementary appendix [file mmc1.pdf]

THE LANCET
Global Health

Supplementary appendix

This appendix formed part of the original submission and has been peer reviewed. We post it as supplied by the authors.

Supplement to: Shakya M, Voysey M, Theiss-Nyland K, et al. Efficacy of typhoid conjugate vaccine in Nepal: final results of a phase 3, randomised, controlled trial. *Lancet Glob Health* 2021; **9:** e1561–68.

Supplementary Appendix

Contents

1.0 Sample size calculation 2

2.0 Inclusion and exclusion criteria 3

2.0 Supplementary Tables and Figures 4

Figure S1. Trial flow diagram 4

Figure S2. Boxplot of Anti-Vi IgA Response at Different Timepoints According to Age Category 5

Figure S3. Boxplot of Anti-Vi IgA Response at Different Timepoints According to Age Category 6

Table S1. Anti-Vi IgA and IgG Levels at Baseline, 28 Days and 18 Months after Randomization in the Immunogenicity Cohort, by Age Group 7

Table S2. Anti-Vi IgA and IgG Levels at Baseline, 28 Days, 18 Months after Randomization in the Immunogenicity Cohort, by Sex 10

Table S3. Fever Presentations and Protective Efficacy of Typhoid Conjugate Vaccine (TCV) 14

Table S4. Hospitalizations, Absenteeism and Protective Efficacy of Typhoid Conjugate Vaccine (TCV) 16

Table S5. Causes of Deaths in the Study Cohort 18

Table S6. Antibiotic susceptibility profile for *S.* Typhi isolates 19

1.0 Sample size calculation

Sample size calculations are based on the following assumptions:

1. An overall incidence of typhoid fever of 85 cases per 100,000 person-years in the entire population, with higher incidence rates in children under 16 years.
2. Age specific incidence rates were determined from the age distribution of typhoid cases in to Kathmandu, from published estimates and from unpublished site-specific surveillance data.
3. A direct effect of vaccination of 75% and an indirect effect of 25% based on mathematical modelling.
4. 25% loss to follow-up per year due to moving out of the area, based on unpublished surveillance data from Lalitpur.

With the above assumptions, the calculated sample size was 17,395 children. To allow for further variation in the assumptions, the total sample size was increased to 20,000 children with 10,000 children in each vaccination arm.

2.0 Inclusion and exclusion criteria

Inclusion Criteria

Participants had to fulfill all of the following criteria to be eligible for enrollment:

- Parents/ legal guardians was willing and competent to provide informed consent. Assent was also be sought for participants 7 years and older.
- Aged between 9 months and under-16 years at the time of the vaccination.
- In good health on the day of the vaccination.
- Parents/ legal guardians confirmed that their child would be willing and would be able to comply with the study requirement including follow-up contact.
- Lived within the study catchment area at the time of vaccination.

Exclusion Criteria

Participants were not enrolled if any of the following criteria applied:

- Participant had knowingly received a typhoid vaccine in the last three years.
- Participant had a known allergy to any of the vaccine components.
- Participant or the parent/legal guardian had any medical or social reasons preventing them from conforming to the study requirements as judged by a medical professional.
- Participant or the parent/legal guardian were planning to move away from the catchment area with the next six months.

3.0 Supplementary Figures and Tables

Figure S1. Trial flow diagram

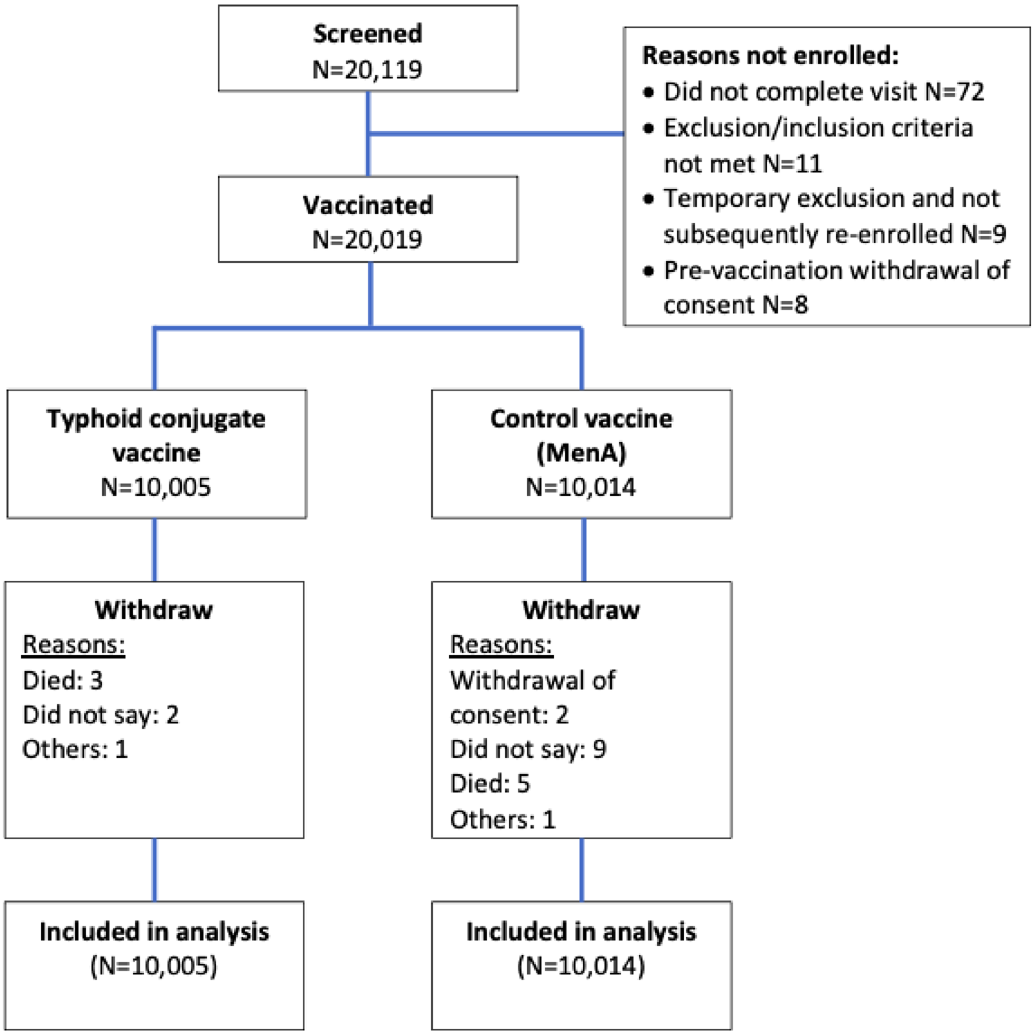

Figure S2. Boxplot of Anti-Vi IgA Response at Different Timepoints According to Age Category.

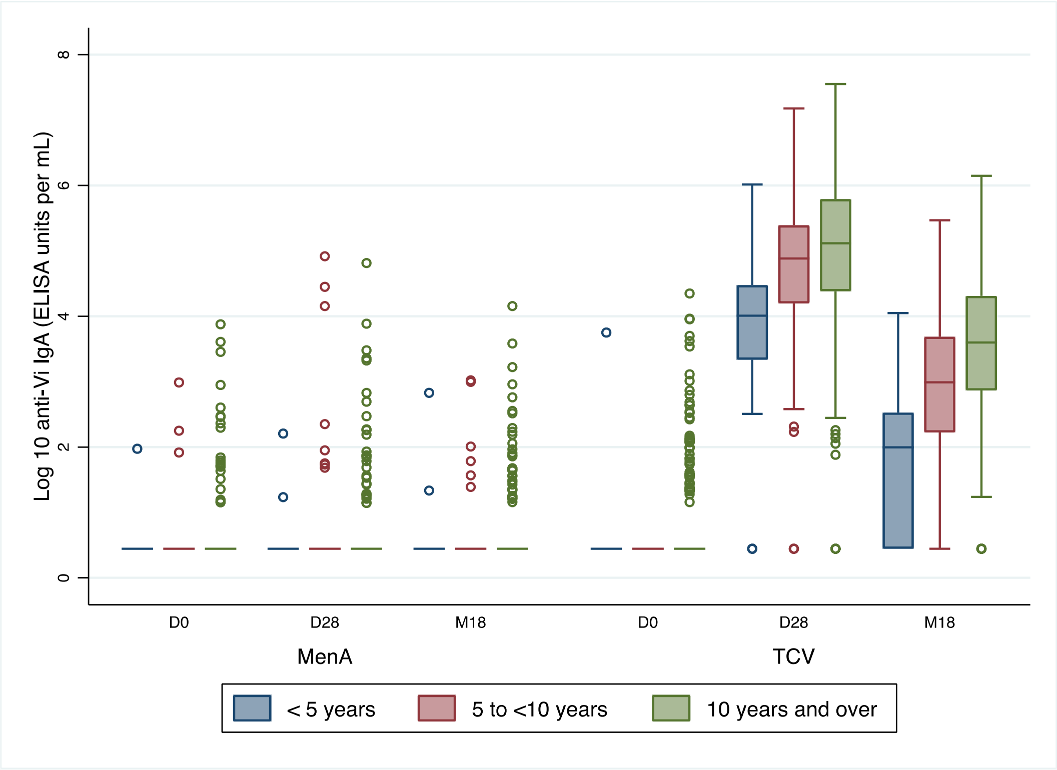

TCV = Typhoid conjugate vaccine. Men A = Group A meningococcal vaccine

Figure S3. Boxplot of Anti-Vi IgG Response at Different Timepoints According to Age Category

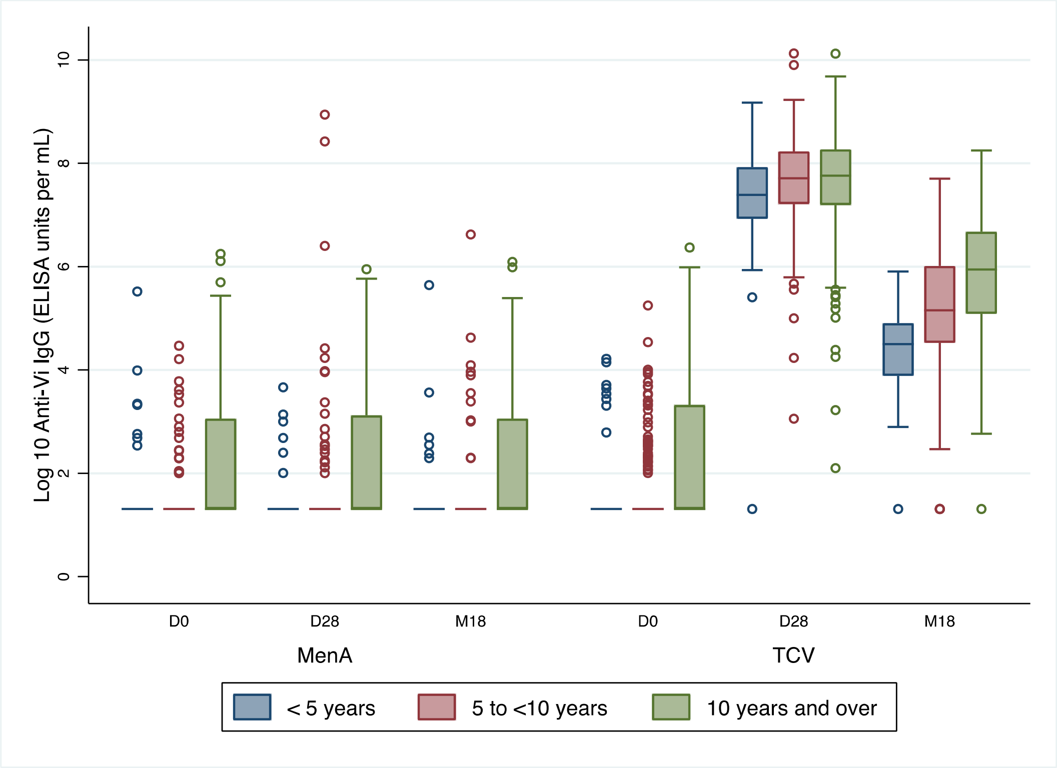

TCV = Typhoid conjugate vaccine. Men A = Group A meningococcal vaccine

Table S1. Anti-Vi IgA and IgG Levels at Baseline, 28 Days, and 18 Months after Randomization in the Immunogenicity Cohort, by Age Group

Trial Group	Day 0	Day 28	Month 18	Period from Day 0 – Day 28	Period from Day 0 – Month 18	Day 0	Day 28	Month 18	Period from Day 0 – Day 28	Period from Day 0 – Month 18	Day 0	Day 28	Month 18	Period from Day 0 – Day 28	Period from Day 0 – Month 18	P-value†
Age Category	Less than 5 years					5 – <10 years					10 years and above					
				4-fold rise	4-fold rise				4-fold rise	4-fold rise				4-fold rise	4-fold rise	
**Anti-Vi IgA Level**																
**TCV**																
Level above lower limit of quantification of the assay – no. of participants/ total no. (%)*	1/55 (1.82)	52/55 (94.55)	56/76 (73.68)	52/55 (94.55)	25/42 (59.52)	0/225 (0.00)	221/225 (98.22)	209/220 (95.00)	221/225 (98.22)	160/182 (87.91)	55/403 (13.65)	397/403 (98.51)	336/343 (97.96)	391/403 (97.02)	294/315 (93.33)	
Geometric mean concentration (95% CI) – EU/ml	1.66 (1.47– 1.89)	46.77 (34.54 – 63.33)	6.02 (4.83 – 7.51)	47.44 (34.38 – 60.50) fold increase	6.22 (4.30 – 8.13) fold increase	1.56 (1.56 – 1.56)	112.0 (97.19 – 129 .08)	18.20 (15.81 – 20.95)	110.84 (97.17 – 124.51) fold increase	21.04 (17.55- 24.55) fold increase	1.96 (1.84 – 2.09)	145.92 (130.45 – 163.23)	35.56 (31.67 – 39.93)	135.43 (121.35 – 149.51) fold increase	34.07 (29.58 – 38.55) fold increase	D0<0.001 D28<0.001 M18<0.001 D0-D28 < 0.001 D0-M18 <0.001
Median (IQR)	1.56 (1.56 – 1.56)	55.17 (28.11 – 87.87)	7.35 (1.56 – 12.52)			1.56 (1.56 – 1.56)	132.08 (66.34 – 219.73)	19.89 (9.25 – 39.92)			1.56 (1.56 – 1.56)	166.87 (80.07 – 327.66)	36.58 (17.57 – 74.60)			
**MenA vaccine**																
Level above lower limit of quantification	1/46 (2.17)	2/46 (4.35)	2/57	0/46 (0.00)	1/35 (2.86)	3/116 (2.59)	8/116 (6.90)	6/110 (5.45)	3/116 (2.59)	2/93 (2.15)	21/218 (9.63)	28/218 (12.84)	33/191 (17.28)	3/218 (1.38)	3/171 (1.75)	

of the assay – no. of participants/ total no. (%)*			(3.51)													
Geometric mean concentration (95% CI) – EU/ml	1.61 (1.51 – 1.72)	1.65 (1.51 – 1.79)	1.65 (1.51 – 1.81)	1.03 (0.98 – 1.09) fold increase	1.32 (0.75-1.90) fold increase	1.64 (1.55 – 1.74)	1.84 (1.62 – 2.10)	1.70 (1.58 – 1.85)	2.59 (0.73 – 4.45) fold increase	1.26 (0.90-1.62) fold increase	1.83 (1.70 – 1.97)	1.93 (1.77 – 2.11)	2.02 (1.84 – 2.21)	1.52 (0.79 – 2.25) fold increase	1.25 (1.08 – 1.44) fold increase	D0=0.0225 D28=0.1002 M18=0.0012 D0-D28 =0.8942 D0-M18 =0.1865
Median (IQR)	1.56 (1.56 – 1.56)	1.56 (1.56 – 1.56)	1.56 (1.56 – 1.56			1.56 (1.56 – 1.56)	1.56 (1.56 – 1.56)	1.56 (1.56 – 1.56			1.56 (1.56 – 1.56)	1.56 (1.56 – 1.56)	1.56 (1.56 – 1.56			
**Anti-Vi IgG Level**																
**TCV**																
Level above lower limit of quantification of the assay – no. of participants/ total no. (%)**	8/99 (8.08)	66/67 (98.51)	75/76 (98.68)	54/55 (98.18)	55/58 (94.83)	44/288 (15.28)	234/234 (100.00)	216/220 (98.18)	225/225 (100.00)	202/209 (96.65)	216/462 (46.75)	408/408 (100.00)	342/343 (99.71)	398/403 (98.76)	316/334 (94.61)	
Geometric mean concentration (95% CI) – EU/ml	4.45 (3.92–5.06)	1488.31 (1160.83 - 1908.17)	81.00 (67.23 – 97.58)	525.20 (403.81 – 646.58) fold increase	28.65 (22.63 – 34.68) fold increase	4.71 (4.38 – 5.08)	2093.13 (1878.03 – 2332.87)	182.29 (156.74 – 212.00)	619.83 (556.98 – 682.67) fold increase	69.39 (58.37 – 80.41) fold increase	10.42 (9.26 – 11.70)	2113.18 (1929.68 – 2314.18)	367.87 (328.92 – 411.43)	431.94 (382.09 – 481.79) fold increase	72.87 (62.50 – 83.25) fold increase	D0 < 0.001 D28=0.0073 M18 < 0.001 D0-D28 < 0.001 D0-M18 <0.001
Median (IQR)	3.7 (3.7 – 3.7)	1619.37 (1018.63 – 2766.08)	89.95 (48.73 – 135.29)			3.7 (3.7 – 3.7)	2227.37 (1353.86 – 3752.75)	173.55 (92.34 – 408.76)			3.7 (3.7 – 27.72)	2342.88 (1327.70 – 3902.43)	382.20 (161.97 – 793.64)			

MenA vaccine																
Level above lower limit of quantification of the assay – no. of participants/ total no. (%)**	7/66 (10.61)	6/49 (12.24)	6/57 (10.53)	0/46 (0.00)	1/47 (2.13)	18/144 (12.50)	19/121 (15.70)	11/110 (10.00)	5/116 (4.31)	3/98 (3.06)	97/250 (38.80)	87/218 (39.91)	79/191 (41.36)	3/218 (1.38)	6/186 (3.23)	
Geometric mean concentration (95% CI) – EU/ml	4.64 (3.88 – 5.57)	4.45 (3.82 – 5.19)	4.51 (3.75 – 5.43)	1.00 (0.90 – 1.10) fold increase	2.60 (-0.62 – 5.83) fold increase	4.50 (4.09 – 4.98)	5.42 (4.37 – 6.71)	4.70 (4.03 – 5.48)	15.54 (-6.01 – 37.09) fold increase	1.64 (0.87 – 2.45) fold increase	8.73 (7.51- 10.15)	8.88 (7.54 – 10.47)	8.90 (7.49 – 10.59)	1.27 (0.91 – 1.64) fold increase	1.19 (0.93 – 1.45) fold increase	D0 < 0.001 D28 < 0.001 M18 < 0.001<br>D0-D28= 0.0572 D0-M18= 0.1461
Median (IQR)	3.7 (3.7 – 3.7)	3.7 (3.7 – 3.7)	3.7 (3.7 – 3.7)			3.7 (3.7 – 3.7)	3.7 (3.7 – 3.7)	3.7 (3.7 – 3.7)			3.7 (3.7 – 22.68)	3.7 (3.7 – 22.68)	3.7 (3.7 – 21.26)			

TCV = Typhoid conjugate vaccine. Men A = Group A meningococcal vaccine

† Kruskal_Wallis test

*The lower limit of quantification was 3.125 EU per millilitre. Values below this limit were substituted with 1.56 EU per millilitre for the analysis.

**The lower limit of quantification was 7.4 EU per millilitre. Values below this limit were substituted with 3.7 EU per millilitre for the analysis.

Table S2. Anti-Vi IgA and IgG Levels at Baseline, 28 Days and 18 Months after Randomization in the Immunogenicity Cohort, by Sex

Trial Group	Day 0	Day 28	18 months	Period from Day 0 – Day 28	Period from Day 0 – 18 months	Day 0	Day 28	18 months	Period from Day 0 – Day 28	Period from Day 0 – 18 months	P-value†
	Male					Female					
**Anti-Vi IgA Level**											
TCV				**4-fold rise**	**4-fold rise**				**4-fold rise**	**4-fold rise**	
Level above lower limit of quantification of the assay – no. of participants/ total no. (%)*	25/356 (7.02)	349/356 (98.03)	311/336 (92.56)	348/356 (97.75)	242/277 (87.36)	31/327 (9.48)	321/327 (98.17)	290/303 (95.71)	316/327 (96.64)	237/262 (90.46)	
Geometric mean concentration (95% CI) – EU/ml	1.73 (1.66 – 1.81)	112.62 (99.72 – 127.19)	19.70 (17.30 – 22.43)	113.38 (101.27 – 124.49) fold increase	25.20 (21.28 – 29.12) fold increase	1.87 (1.74 – 1.99)	133.18 (117.49 – 150.97)	26.96 (23.56 – 30.86)	127.72 (112.43 – 143.01) fold increase	29.93 (25.46 – 34.40) fold increase	D0=0.0.2098 D28=0.0611 M18=0.0016 D0-D28= 0.2516 D0-M18= 0.0331
Median (IQR)	1.56 (1.56 – 1.56)	130.69 (63.06 – 263.66)	20.63 (9.38 – 45.19)			1.56 (1.56 – 1.56)	148.04 (72.13 – 278.13)	27.14 (12.67 – 57.30)			
MenA vaccine											

Level above lower limit of quantification of the assay – no. of participants/ total no. (%)*	9/192 (4.69)	16/192 (8.33)	15/181 (8.29)	2/192 (1.04)	4/151 (2.65)	16/188 (8.51)	22/188 (11.70)	26/177 (14.69)	4/188 (2.13)	2/148 (1.35)	
Geometric mean concentration (95% CI) – EU/ml	1.67 (1.59 – 1.74)	1.74 (1.64 – 1.85)	1.79 (1.66 – 1.93)	1.28 (0.86 – 1.69) fold increase	1.30 (1.05 – 1.55) fold increase	1.83 (1.68 – 1.98)	2.01 (1.79 – 2.25)	1.92 (1.77 – 2.09)	2.31 (0.95 – 3.66) fold increase	1.23 (1.12 – 1.45) fold increase	D0=0.1243 D28=0.2117 M18=0.0676 D0-D28= 0.7385 D0-M18= 0.6716
Median (IQR)	1.56 (1.56 – 1.56)	1.56 (1.56 – 1.56)	1.56 (1.56 – 1.56)			1.56 (1.56 – 1.56)	1.56 (1.56 – 1.56)	1.56 (1.56 – 1.56)			
**Anti-Vi IgG Level**											
TCV											
Level above lower limit of quantification of the assay – no. of participants/ total no. (%)**	136/450 (30.22)	372/373 (99.73)	332/336 (98.81)	352/356 (98.88)	302/314 (96.18)	132/399 (33.08)	336/336 (100.00)	301/303 (99.34)	325/327 (99.39)	271/287 (94.43)	

Geometric mean concentration (95% CI) – EU/ml	7.08 (6.39 – 7.83)	1920.49 (1747.09 – 2111.10)	213.62 (188.23 – 242.43)	482.33 (431.03 – 533.63) fold increase	60.80 (52.26 – 69.35)	7.37 (6.59 – 8.23)	2176.67 (1976.24 – 2397.42)	276.19 (242.30 – 314.82)	522.04 (466.42 – 577.65) fold increase	74.60 (63.31 – 85.89)	D0=0.5106 D28=0.1021 M18=0.0080 D0 – D28= 0.3166 D0 – M18= 0.0305
Median (IQR)	3.7 (3.7 – 13.43)	2156.67 (1269.61 – 3606.48)	200.32 (97.51 – 528.86)			3.7 (3.7 – 13.47)	2311.84 (1327.70 – 3886.78)	267.72 (123.72 – 670.13)			
MenA vaccine											
Level above lower limit of quantification of the assay – no. of participants/ total no. (%)**	62/236 (26.27)	56/196 (28.57)	41/181 (22.65)	3/192 (1.56)	4/166 (2.41)	60/244 (26.79)	56/192 (29.17)	55/177 (31.07)	5/188 (2.66)	6/165 (3.64)	
Geometric mean concentration (95% CI) – EU/ml	6.18 (5.46 – 6.99)	6.50 (5.58 – 7.57)	6.01 (5.17 – 6.98)	2.56 (-0.14 – 5.26) fold increase	1.29 (0.89 – 1.69) fold increase	6.82 (5.88 – 7.90)	7.50 (6.25 – 9.00)	7.19 (6.08 – 8.51)	8.69 (-4.30 – 21.69) fold increase	1.76 (0.79 – 2.73) fold increase	D0=0.6142 D28= 0.5547 M18=0.0736 D0 – D28= 0.8198

											D0 – M18= 0.8458
Median (IQR)	3.7 (3.7 – 8.53)	3.7 (3.7 – 9.47)	3.7 (3.7 – 3.7)			3.7 (3.7 – 9.41)	3.7 (3.7 – 14.12)	3.7 (3.7 – 11.91)			

TCV = Typhoid conjugate vaccine. Men A = Group A meningococcal vaccine

† Mann-Whitney U test

*The lower limit of quantification was 3.125 EU per millilitre. Values below this limit were substituted with 1.56 EU per millilitre for the analysis.

**The lower limit of quantification was 7.4 EU per millilitre. Values below this limit were substituted with 3.7 EU per millilitre for the analysis.

Table S3. Fever Presentations and Protective Efficacy of Typhoid Conjugate Vaccine (TCV).

Outcome	Number of fevers in TCV group (N=10005 participants)	Number of fevers in Men A group (N=10014 participants)	Vaccine Efficacy (95% CI)	P-value
Fever presentations at clinics/hospital				
**Number of presentations with fever of any duration**	2390	2408	0.7% (-5.1%, 6.2%)	0.8093
**Number of fevers of ≥2 days, or a temperature of 38 degrees C at presentation**	2352	2366	0.5% (-5.3%, 6.1%)	0.8529
**Number of fevers of ≥3 days, or a temperature of 38 degrees C at presentation**	1980	1968	-1.1% (-7.7%, 5.0%)	0.7259
**Clinically suspected typhoid at fever presentation (clinician recorded)**	417	415	-0.5% (-15.4%, 12.4%)	0.9386
Self-reported fevers from follow up phone calls				
**Number of self-reported fevers via phone or follow up contact**	4816	4766	-1.1% (-5.35%, 2.9%)	0.5914
**Number of self-reported fevers that did not result in visit to health care provider**	1258	1267	0.6% (-7.5%, 8.2%)	0.8684
**Number of self-reported fevers that resulted in pharmacy visit**	759	780	2.6% (-7.7%, 12.0%)	0.5998

**Duration of self-reported fever**<br>**Median days per fever episode [IQR]**	4305 fevers<br>3 [2, 4]<br>N=3915 persons with fever	4287 fevers<br>3 [2, 4]<br>N=3922 persons with fever		
Number of days of self-reported fever per person	N=2886<br>Sum= 15,748<br>4 [2, 7]	N=2863<br>Sum=15,778<br>4 [2, 7]		
**Self-reported suspected/confirmed typhoid fevers from phone call follow up**				
**Number of clinically suspected typhoid fevers - self-reported**	96	120	19.9%<br>(-5.6%, 39.4%)	0.1039
**Number of clinically diagnosed typhoid fevers - self-reported**	31	71	56.3%<br>(32.5%, 72.3%)	< 0.001
**Duration of self-reported typhoid fever, median days per fever episode [IQR]**	31<br>6 [4,8]	68<br>7.5 [5, 11.5]		0.0084
Total number of days of self-reported confirmed typhoid fever	218	624		

TCV = Typhoid conjugate vaccine. Men A = Group A meningococcal vaccine

Table S4. Hospitalizations, Absenteeism and Protective Efficacy of Typhoid Conjugate Vaccine (TCV).

Outcome	TCV (N=10005)	Men A (N=10014)	Vaccine Efficacy (95% CI)	p value
**Hospitalisations**				
**Hospitalisation (all cause) *[self-report from phone call follow up]***	149	141	-5.7% (-34.1%, 16.6%)	0.6359
**Length of hospital stay (all cause) *[self-report from phone call follow up]***	138<br>5 [3,7]	127<br>5 [3,7]		0.3339
**Length of hospital stay (all cause) *[SAE]***	178<br>4[3,7]	194<br>5[3,7]		0.2926
Hospitalisation for fever [SAE]	148	139	-9.1% (-37.7%, 13.6%)	0.4517
Hospitalisation for typhoid (suspected or clinical diagnosis) *[SAE]*	16	18	11.1% (-84.8%, 57.6%)	0.7370
Hospitalisation for typhoid (confirmed BC+) *[SAEs]*	2	7	71.4% (-50.1%, 97.1%)	0.1095
**Death (all cause)**	3	5	40.0% (-208.6%, 90.6%)	0.5083
**Days missed from school/work**				
**Days of school missed, median number days per person [IQR]**	N= 1928 pts<br>4 [2, 7]	N=1859 pts<br>4 [2, 7]		0.8283*
**Days of work missed, median number of days per person [IQR]**	N=187<br>3 [2, 7]	N=215<br>4 [2, 7]		0.5882*

**Days missed school for self-reported typhoid fever, median number days *per episode* [IQR]**	N=28<br>10 [5, 14.5]	N= 61<br>10 [6, 15]		0.6640*
**Days missed work for self-reported typhoid fever, median number days *per episode* [IQR]**	N=7<br>6[2,14]	N=14<br>7[6,14]		0.4621*

TCV = Typhoid conjugate vaccine. Men A = Group A meningococcal vaccine

*Wilcoxon rank sum test

Table S5. Causes of Deaths in the Study Cohort

Vaccine Arm	Age at enrollment (years)	Time after vaccination (months)	Diagnosis
MenA	3.1	7	Multiple organ dysfunction syndrome with severe pneumonia with staphylococcal sepsis
MenA	8.1	14	Acute kidney injury with pulmonary edema
MenA	1.3	14	Pneumonia
MenA	13.6	16	Known intestinal malignancy
TCV	13.8	16	? Homicide, details not provided by the family
MenA	8.1	18	Head injury
TCV	0.8	23	Fall from height
TCV	1.6	NA	Suspected death, no information available from family

Table S6. Antibiotic susceptibility profile for *S*. Typhi isolates

	MenA		TCV	
	**Susceptible**	**Non-susceptible**	**Susceptible**	**Non-susceptible**
**Amoxycillin**	53 (100%)		11 (100%)	
**Chloramphenicol**	53 (100%)		11 (100%)	
**Trimethoprim-sulfamethoxazole**	53 (100%)		11 (100%)	
**Ceftriaxone**	53 (100%)		11 (100%)	
**Ciprofloxacin**	10 (19%)	43* (81%)	1 (9%)	10* (91%)
**Azithromycin**	52 (98%)	1** (2%)	10 (91%)	1** (9%)

*MIC>0.06mg/L; **MIC>16mg/L

TCV = Typhoid conjugate vaccine. Men A = Group A meningococcal vaccine
